# Endothelium-Dependent Relaxation Effect of *Apocynum venetum* Leaf Extract via Src/PI3K/Akt Signalling Pathway

**DOI:** 10.3390/nu7075220

**Published:** 2015-06-30

**Authors:** Yeh Siang Lau, Wei Chih Ling, Dharmani Murugan, Chiu Yin Kwan, Mohd Rais Mustafa

**Affiliations:** 1 Department of Pharmacology, Faculty of Medicine, University of Malaya, Kuala Lumpur 50603, Malaysia; E-Mails: lauyehsiang@um.edu.my (Y.S.L.); tze_chih89@yahoo.com (W.C.L.); dharmani79@um.edu.my (D.M.); 2 Graduate Institutes of Basic Medical Sciences and Vascular Biology Group, China Medical University and Hospitals, Taichung 40402, Taiwan; E-Mail: kwancy@mail.cmu.edu.tw

**Keywords:** *Apocynum venetum*, nitric oxide, endothelium, vasorelaxation, antihypertensive medicinal herb

## Abstract

Botanical herbs are consumed globally not only as an essential diet but also as medicines or as functional/recreational food supplements. The extract of the *Apocynum venetum* leaves (AVLE), also known as Luobuma, exerts its antihypertensive effect via dilating the blood vessels in an endothelium- and concentration-dependent manner with optimal effect seen at as low as 10 µg/mL. A commercial Luoboma “antihypertensive tea” is available commercially in the western province of China. The present study seeks to investigate the underlying cellular mechanisms of the nitric oxide (NO)-releasing property of AVLE in rat aortas and human umbilical vein endothelial cells (HUVECs). Endothelium-dependent relaxation induced by AVLE was assessed in organ chambers in the presence or absence of polyethyleneglycol catalase (PP2, 20 µM; inhibitor of Src kinase), wortmannin (30 nM) and LY294002 (20 µM; PI3 (phosphatidylinositol3)-Kinase inhibitor), N^G^-nitro-l-arginine (L-NAME, 100 µM; endothelial NO synthase inhibitor (eNOS)) and ODQ (1 µM; soluble guanylyl cyclase inhibitor). Total nitrite and nitrate (NOx) level and protein expression of p-Akt and p-eNOS were measured. AVLE-induced endothelium-dependent relaxation was reduced by PP2, wortmannin and LY294002 and abolished by L-NAME and ODQ. AVLE significantly increased total NO_x_ level in rat aortas and in HUVECs compared to control. It also instigated phosphorylation of Akt and eNOS in cultured HUVECs in a concentration-dependent manner and this was markedly suppressed by PP2, wortmannin and LY294002. AVLE also inhibited superoxide generated from both NADPH oxidase and xanthine/xanthine oxidase system. Taken together, AVLE causes endothelium-dependent NO mediated relaxations of rat aortas through Src/PI3K/Akt dependent NO signalling pathway and possesses superoxide scavenging activity.

## 1. Introduction

Hypertension, a cardiovascular disease with increased arterial pressure and increased vascular resistance, is a major contributor to morbidity and mortality [1]. Increased sodium-retaining hormones and sympathetic nervous system activity, overproduction of endothelium-derived contracting factors (EDCFs), and deficiencies of vasodilators such as nitric oxide (NO) are among the pathophysiological factors implicated in hypertension [1]. Hypertension is strongly correlated with increased oxidative stress, vascular remodelling and endothelial dysfunction, which collectively contribute to the development of hypertension and its complications [1,2].

Traditional Chinese Medicinal (TCM) herbs have been used for more than thousands of years and its role for treating hypertension remains strong with increasing interests for research. In addition to reducing blood pressure, TCM herbs are often used for the relief of symptoms related to cardiac problems and stroke, removing blood stagnation, reversing vascular remodelling and improving endothelium-dependent dilation of blood vessels [3,4,5]. In general, they act by improving the circulation and eliciting vascular relaxant properties. Some of them act directly on the vascular smooth muscle cells via a variety of signalling mechanisms or indirectly via an endothelium-dependent manner, mostly involving the release of NO [6]. TCM herbs contain a large amount of polyphenols, including flavonoids and non-flavonoids, which have cardiovascular protective actions such as eliciting endothelium-dependent vasorelaxation through the increase in release of NO in endothelial cells [7,8]. Polyphenols are found in a variety of vegetables, fruits and beverages such as red wine, green tea, grape juice and grape skin. Several studies have indicated that endothelium-dependent relaxation induced by polyphenols is mainly mediated by activation of the phosphatidylinositol-3 (PI3) kinase/Akt signalling pathway [9,10,11].

The dried leaves of *Apocynum venetum* Linnaeus, a plant indigenous to Xinjiang, a western province of China, are commercialized locally as “antihypertensive tea”. Indeed, the leaf extract of *Apocynum venetum*, referred to as AVLE hereafter, has been shown to lower blood pressure *in vivo* and cause *in vitro* vasodilatation of rat aortic and mesenteric arterial rings [12,13]. The leaves are rich in ash elements, minerals and flavonoids and have been shown to possess antioxidant, anti-lipid peroxidation, anti-depressant, anti-anxiety, antihypertensive, anti-hyperlipidaemic, hepatoprotective, cardiotonic and diuretic effects [6,12,14,15,16,17]. Kwan *et al.* demonstrated that the endothelium-dependent relaxation induced by AVLE was potent, with maximal relaxation occurring at 10 µg/mL, and could be inhibited by NO synthase inhibitor, N^G^-nitro-l-arginine (L-NAME) and K^+^ channel blocker, tetramethylammonia. Unlike carbachol-induced relaxation, which is a standard indicator for endothelium-dependent and NO-mediated events, AVLE-induced vasodilatation was long-lasting and persistent even after repeated washout of AVLE. It has been proposed that this effect could be due to its nitric oxide releasing and superoxide-scavenging properties. However, there has been no study of its cellular or molecular mechanism of actions. The increasing popularity of “Luobuma tea” to treat hypertension dictates the need to explore underlying mechanisms of actions of AVLE which may mediate its vasorelaxation effect. In the present study, we hypothesize that AVLE elicits NO production via Src/PI3K/Akt signalling pathway to cause vasodilaton of the rat thoracic aorta.

## 2. Experimental Section

### 2.1. Materials

*Apocynum venetum* leaf extracts (AVLE) were extracted from the dried leaves of *Apocynum venetum* Linn. It contained not less than 4.0% of hyperoside and isoquercitrin, calculated on the dried basis. AVLE was obtained from Tokiwa Phytochemical Company (Tokyo, Japan) and commercially prepared as a standard water-soluble extract in brown-colored powder form under the trade name VENETRON. Sodium-HEPES, acetylcholine chloride, phenylephrine hydrochloride, angiotensin II, aspergillus nitrate reductase, Griess reagent, bis-N-methylacridinium nitrate (lucigenin), diethyldithiocarbamate acid (DETCA), diphenyliodonium (DPI), and β-nicotinamide adenine dinucleotide phosphate (NADPH) were purchased from Sigma-Aldrich (St. Louis, MO, USA). Sodium nitroprusside and Krebs salts (BDH, Poole Dorset, UK). Quercetin and DPI were dissolved in DMSO. Lucigenin, NADPH and DETCA were dissolved in Kreb-HEPES buffer and all other chemicals in distilled water.

### 2.2. Animals

Male Sprague Dawley (SD, 10 weeks old) rats were purchased from an animal experimental unit (AEU), University of Malaya. All experimental procedures were subjected to the University of Malaya Animal Experimentation Ethics Committee approval. All rats were housed in a well-ventilated room at 22 ± 2 °C, 30%–40% humidity and had free access to standard rat chow (Altromin Spezialfutter GmbH & Co., Lage, Germany) and tap water *ad libitum*.

### 2.3. Aortic Ring Preparation and Functional Study

The rats were sacrificed by CO_2_ inhalation. The descending thoracic aorta was then isolated through the chest. The aorta was cut into the 3–4 mm long segment and placed into the Krebs physiological salt solution (KPSS) [composition: NaCl 118.2, NaHCO_3_ 25, KCl 4.7, KH_2_PO_4_ 1.2, MgSO_4_·7H_2_O 1.2, glucose 11.7, and CaCl_2_·2H_2_O 2.5 mM] for isometric tension measurement or in ice-cold Krebs-HEPES buffer [composition: NaCl 99.0, NaHCO_3_ 25, KCl 4.7, KH_2_PO_4_ 1.0, MgSO_4_·7H_2_O 1.2, glucose 11.0, CaCl_2_·2H_2_O 2.5 and Na-HEPES 20.0 mM] for superoxide generation assay following the removal of fat and connective tissues. The fresh aortic rings were maintained at 37 °C and the rings were connected to isometric force-displacement transducers (Grass Instrument Co, Quincy, MA, USA), and the output was amplified and recorded continuously using the PowerLab LabChart 6.0 recording system (AD Instruments, NSW, Australia). The rings were equilibrated for 45 min under 1.0 g resting tension before being stimulated with a single high KCl solution (high K^+^, 60 mM) for 15 min. The presence of endothelium in the aortic segments was assessed by inducing relaxation with a single exposure to acetylcholine (ACh, 10 µM) following a phenylephrine (1 µM) pre-contraction. To study the effect of AVLE-induced NO-mediated relaxation, AVLE (100 ng/mL- 100 µg/mL) was added concentration dependently to the rings pre-contracted with phenylephrine. In another set of experiments, the isolated aortic rings were incubated in the presence or absence of different pharmacological inhibitors which includes polyethyleneglycol catalase (PP2, 20 µM), inhibitor of Src kinase; wortmannin (30 nM) and, LY294002 (20 µM), PI3-Kinase (PI3K) inhibitor; N^G^-nitro-l-arginine (L-NAME, 100 µM), endothelial NO synthase inhibitor (eNOS) and ODQ (1 µM), soluble guanylyl cyclase inhibitor) for 30 min before AVLE-induced vasorelaxation was performed.

### 2.4. Measurement of Vascular Superoxide Production

The bioavailability of NO in the vascular tissues is dependent upon the amount of free radical presence in the vascular cell, particularly, the superoxide anion, which reacts rapidly with NO to form peroxynitrite and thereby decreasing NO bioavailability. Hence, the following experiments were aimed at investigating the superoxide anion scavenging property of AVLE using Lucigenin-enhanced chemiluminescence and dihydroethidium (DHE) dye.

#### 2.4.1. Measurement of NADPH-Mediated Superoxide Production in Aortic Ring and Cryostat Section

Lucigenin-enhanced chemiluminescence and DHE were used to estimate the vascular superoxide (O_2_^−^) production as described previously [18]. The segment of aortic rings were pre-incubated in 2 mL of Krebs-HEPES buffer with either AVLE extract in a concentration manner (0, 0.3, 1, 10, 30, and 100 µg/mL) or diphenylene iodonium (DPI, 5 µM), an inhibitor of NADPH oxidase in the presence of diethylthiocarbamic acid (DETCA, 1 mM) to inactivate superoxide dismutase and β-nicotinamide adenine dinucleotide phosphate (NADPH, 0.1 mM) as a substrate for NADPH oxidase. The reaction mixtures were incubated for 45 min at 37 °C. Prior to measurement of chemiluminescence reading, 96-well Optiplate with its wells filled with 300 µL of Krebs-HEPES buffer containing lucigenin (5 μM) and NADPH (0.1 mM) was loaded into the Hidex plate CHAMELEON™ V (Turku, Finland) in luminescent detection mode to measure the background photo emission over 20 min. At the end of the incubation period, tissues were washed and transferred to each appropriate well of the plates and the measurement of photo emission was carried out over 20 min to measure O_2_^−^ production. Tissues were dried for 48 h at 65 °C to allow O_2_^−^ production to be normalized to dry tissue weight. The data were expressed as average counts per mg of vessel dry weight.

For DHE fluorescence detection, the aortic rings were treated similarly as above. At the end of incubation, the aortic segments from all treated groups were frozen and cut into 10 μm-thick sections with a microtome and placed on a slide. DHE (5 μM diluted normal physiological saline solution (NPSS, composition in mM: NaCl 140, KCl 5, CaCl_2_ 1, MgCl_2_ 1, glucose 10 and HEPES 5) was added to each tissue section, and incubated with 15 min at 37 °C. Images were scanned with fluorescence microscope (Leica Microsystems) and the fluorescence intensity was quantified using Leica LAS-AF software versions 2.6.0 (Leica Microsystems).

#### 2.4.2. Measurement of X/XO Superoxide Production

One of the major sources of a superoxide generating enzyme system is through the oxidation of substrate xanthine (X) by xanthine oxidase (XO), which, in turn, the reduced form of XO donates an electron to the molecule oxygen to yield O_2_^−^, therefore XO returns to its oxidized form [19]. The XO catalyzed superoxide production was determined as described by Fang *et al.* [19]. The superoxide radical scavenging activity of AVLE extract was measured by lucigenin-enhanced chemiluminescence which the reaction mixtures containing of 10 μM lucigenin, 200 mU/mL XO, and AVLE extract (0, 0.3, 1, 10, 30, and 100 µg/mL) in 10 mM sodium phosphate buffer (pH 7.4). The 100 μM xanthine was added immediately before the measurement in order to start the reaction. The reaction were measured by using a luminescence microplate reader, Hidex plate CHAMELEON™ V (Finland). Enzyme superoxide dismutase (SOD, 100 U/mL) was added into the system as a positive control to scavenge the O_2_^−^ from the reaction of xanthine/xanthine oxidase.

### 2.5. Culture of Human Umbilical Vein Endothelial Cells

Human umbilical vein endothelial cells (HUVECs) were purchased from ScienCell Research Laboratories (Carlsbad, CA, USA) and cultured in endothelial cell medium (ECM) supplemented with 5% FBS, 1% endothelial cell growth supplement, and 1% of penicillin-streptomycin (ScienCell Research Laboratories) at 37 °C in a humidified atmosphere containing 5% CO_2_ and were used in passages 5–10. All the experiments were performed when the cells reached 80% confluence. The cells were seeded on 6-well plates and exposed to serum-free culture medium for 4 h before treatment.

### 2.6. Determination of the Phosphorylation Level of Akt and eNOS

The cells were treated with AVLE in a time-dependent (0, 5, 10, 15, 20 and 30 min) and concentration-dependent (0.3, 1 and 10 µg/mL, 30 min) manner. The NO releasing effect of AVLE was also investigated in the co-treatment of AVLE and PP2, wortmannin or LY294002. At the end of incubation, the cells were harvested in ice-cold 1X RIPA buffer containing (leupeptin 1 μg/mL, aprotonin 5 μg/mL, PMSF 100 μg/mL, sodium orthovanadate 1 mM, EGTA 1 mM, EDTA 1 mM, NaF 1 mM, and β-glycerolphosphate 2 mg/mL). The lysates were centrifuged at 15,000× *g* for 30 min and supernatant was collected for Western blotting. Protein concentrations of the supernatant were determined by using modified Lowry assay (Bio-Rad Laboratories, Hercules, CA, USA). Total protein concentration of 15–20 μg for each lane was separated in 7.5% sodium dodecyl sulphate (SDS)-polyacrylamide gel and then transferred to an immobilon-P polyvinylidene difluoride membrane (Millipore, Billerica, MA, USA) at 100 V for 120 min. The blots were incubated with 3% bovine serum albumin (BSA) in Tris-buffered saline containing 0.1% Tween 20 (TBS) at room temperature with gentle shaking to block the non-specific binding. At the end of incubation, the blots were incubated with either primary mouse monoclonal antibody of Akt (1:1000, Santa Cruz, CA, USA), eNOS (1:500, BD Transduction Laboratory, Oxford, UK), primary rabbit polyclonal antibody of p-Akt^ser473^ (1:500, Cell Signalling Technology, USA), and p-eNOS^ser1177^ (1:250, Abcam, Cambridge, UK) overnight at 4 °C. The membranes were washed three times and incubated with the respective secondary antibodies conjugated to horseradish peroxidise for 2 h at room temperature before developed with Amersham™ ECL prime Western Blotting detection reagent (Amersham, Bukinghamshire, UK) using x-ray film. The images were scanned and densitometric analysis was performed using Quantity One 1D analysis software. The respective protein expression levels were normalized to housekeeping protein β-actin.

### 2.7. Total Nitrite and Nitrate Detection

Previous studies have indicated that AVLE exerts its antihypertensive action by dilating the blood vessels in an endothelium-dependent and concentration-dependent manner. Therefore, NO product from vascular tissue and cell lysate were measured to determine the role of NO-mediated relaxation in isolated aortic rings in the presence or absence of AVLE and inhibitors. Measurement of total nitrate/nitrite level was measured using Nitrate/Nitrite Colorimetric Assay Kit (Cayman Chemical Company, Ann Arbor, MI, USA). Absorbance was measured at 540 nm using Hidex plate CHAMELEON™ V (Turku, Finland). Standard curve was generated using known concentrations of sodium nitrite. Results were expressed in µM/mg protein.

### 2.8. Statistical Analysis

All results are presented as mean ± standard error of mean (SEM) and n indicates the number of rats used. Concentration-response curves were fitted to a sigmoidal curve using a nonlinear regression for each group. Statistical significance was determined using Student’s *t*-test for unpaired observations and the one-way analysis of variance (ANOVA) followed by Bonferroni’s multiple comparison test for multiple value comparison (GraphPad Prism version 5, San Diego, CA, USA). A value of *p* < 0.05 was considered statistically significant.

## 3. Results

### 3.1. AVLE Induces Endothelium-Dependent Relaxation in Rat Aortas

**Figure 1:**
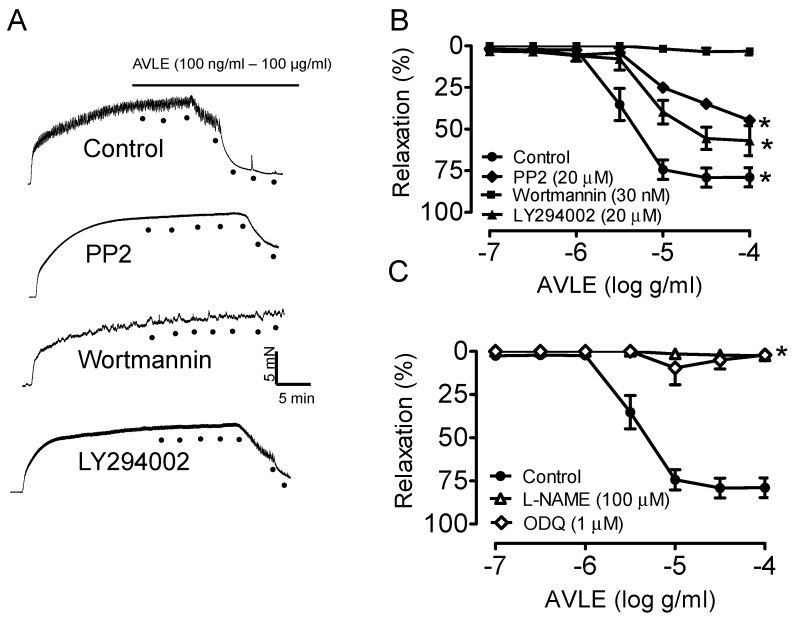
AVLE concentration-dependently (100 ng/mL–100 µg/mL) induced relaxation in isolated rat aortas. (**A**, **B**) Representative traces and graph showing the relaxation induced by AVLE was markedly decreased by inhibitor of Src kinase (PP2, 20 µM); and PI3-Kinase inhibitors (wortmannin, 30 nM and LY294002, 20 µM) (**C**) while eNOS inhibitor (L-NAME, 100 μM,) and soluble guanylate cyclase inhibitor (ODQ, 1 μM) abolished the relaxation response by AVLE. Results are means ± SEM (*n* = 5–6). * *p* < 0.05 compared to control.

Figure 1A,B shows that AVLE caused concentration-dependent (100 ng/mL–100 µg/mL) relaxation in isolated aortic ring. Relaxation to AVLE was inhibited by the Src kinase, PP2 and PI3-Kinase inhibitors, wortmannin and LY294002. The relaxation was also significantly reduced by an eNOS inhibitor, L-NAME and inhibitor of sGC, ODQ (Figure 1C).

### 3.2. AVLE Increases Total Nitrite and Nitrate (NOx) Levels in Rat Aortas

**Figure 2:**
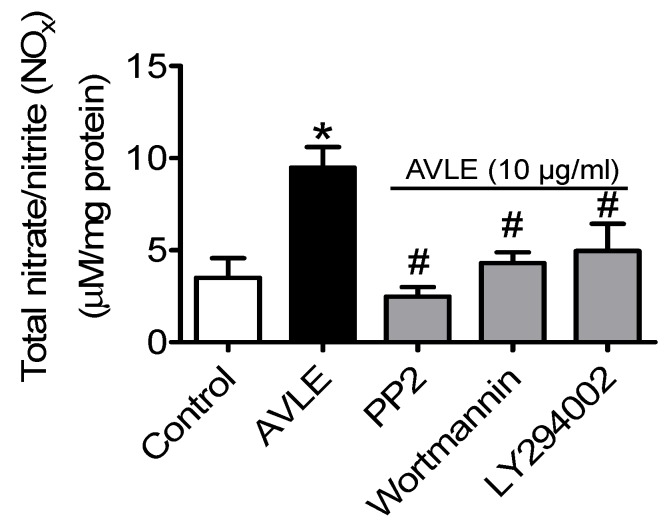
AVLE at 10 µg/mL increased NO production in rat aortas. The NO released induced by AVLE was markedly decreased by inhibitor of Src kinase, PP2 (20 µM) and PI3-Kinase inhibitors, wortmannin (30 nM) and LY294002 (20 µM). Results are means ± SEM (*n* = 5–6). * *p* < 0.05 compared to control, # *p* < 0.05 compared to AVLE.

Total NO metabolites (nitrite and nitrate) level was significantly increased compared to control. The elevated level of NO induced by AVLE was remarkably inhibited by PP2, wortmannin and LY294002 (Figure 2).

### 3.3. AVLE Increases Phosphorylated Akt, eNOS and NO Production in HUVECs

**Figure 3:**
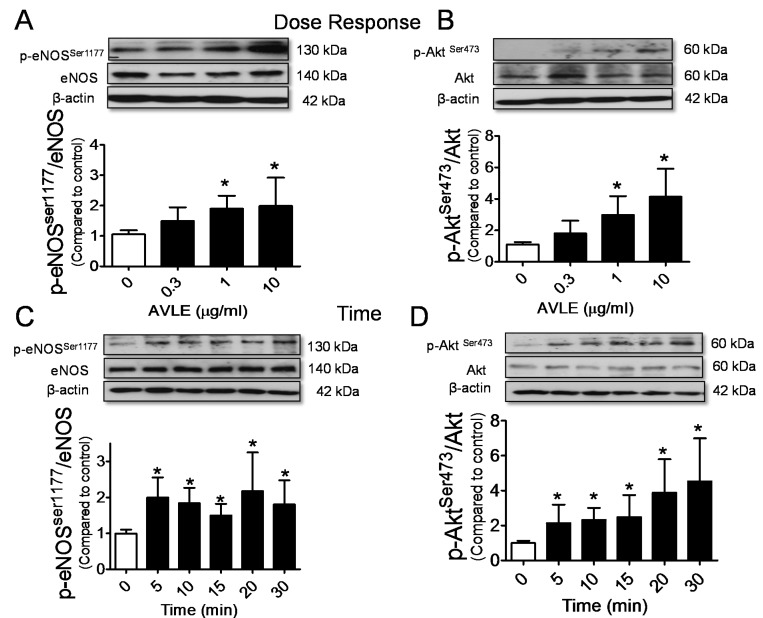
AVLE upregulated phosphorylated eNOS (**A**) and Akt (**B**) protein expression in a concentration-dependent manner (0, 0.3, 1 and 10 µg/mL) in HUVECs. AVLE (10 µg/mL) steadily upregulated phosphorylated eNOS (**C**) and Akt (**D**) protein expression in HUVEC in interval 30 min time points. Results are means ± SEM (*n* = 3). * *p* < 0.05 compared to control.

**Figure 4:**
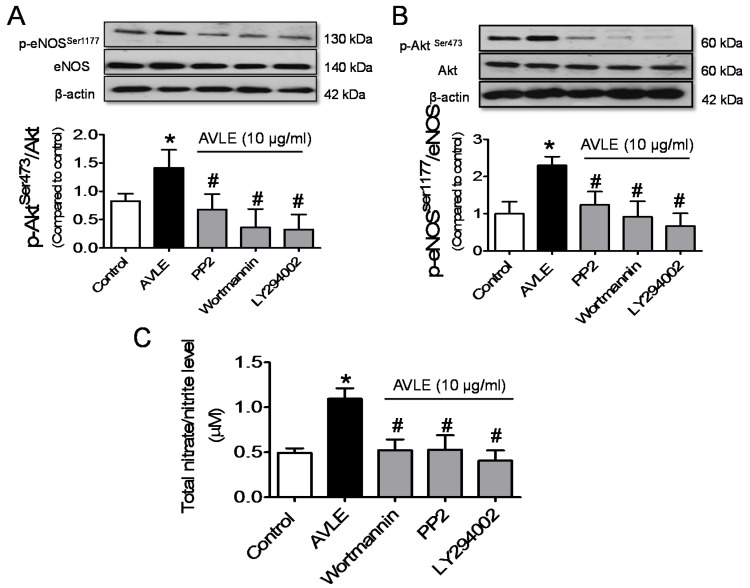
Pre-exposure with inhibitor of Src kinase, PP2 (20 µM) and PI3-Kinase inhibitors wortmannin (30 nM) and LY294002 (20 µM) for 30 min significantly down-regulated p-eNOS^Ser1177^ (**A**); and p-Akt^Ser473^ (**B**) protein expression; and (**C**) total nitrate/nitrite (NO_x_) level induced by AVLE (10 µg/mL, 30 min) in HUVECs. Results are means ± SEM (*n* = 3–5). * *p* < 0.05 compared to control, # *p* < 0.05 compared to AVLE.

AVLE concentration dependently (0.3, 1 and 10 µg/mL) increased phosphorylation of Akt at ser473 (p-Akt^ser473^) and eNOS at ser1177 (p-eNOS^ser1177^) in cultured HUVECs (Figure 3A,B). Figure 3C,D shows the levels of p-Akt^ser473^ and p-eNOS^ser1177^ were significantly increased by AVLE (10 µg/mL) in 5 min and the activity was steadily maintained until the 30 min mark. No significant differences were observed in the elevated level of p-Akt^ser473^ and p-eNOS^ser1177^ stimulated by AVLE at the interval time of 30 min. AVLE-induced phosphorylation of Akt, eNOS and NO level were markedly reduced by Src kinase inhibitor, PP2 and PI3-kinase inhibitors, wortmannin and LY294002 (Figure 4).

### 3.4. AVLE Inhibits Production of Superoxide Anion in Rat Aortas

**Figure 5:**
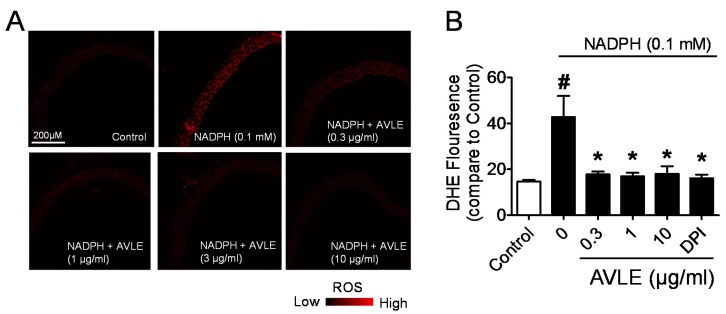

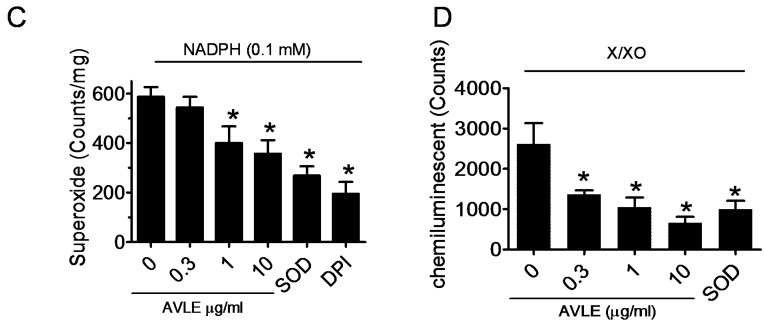
AVLE reduced superoxide generation in both cell and cell-free-mediated superoxide generation systems. The effect of AVLE (0.3, 1 and 10 µg/mL) on (**A**, **B**) superoxide generation from aortic rings using DHE and (**C**) lucigenin-enhanced chemiluminescence in the presence of 0.1 mM NADPH. Diphenyleneiodonium (DPI, 5 µM) was used as NADPH oxidase inhibitor. (**D**) Effect of AVLE and superoxide scavenger, SOD on the superoxide production by X/XO generating system determined by lucigenin-enhanced chemiluminescence. Results are means ± SEM (*n* = 3–5). # *p* < 0.05 compared to control, * *p* < 0.05 compared to zero concentration of AVLE.

AVLE (0.3, 1 and 10 µg/mL) concentration dependently inhibits superoxide produced from NADPH oxidase (Figure 5A–C) and X/XO system (Figure 5D). In this experiment, the effect of DPI, a potent inhibitor of NADPH oxidase, which is one major sources of superoxide generator, was chosen as one positive control. DPI markedly inhibited the *in vitro* production of superoxide from the aortic rings and the effect of AVLE is as potent as DPI (Figure 5A–C). In the cell-free-mediated superoxide generation system, we further demonstrated that AVLE inhibited the enzymatic production of superoxide production in the X/XO cell-free system. Figure 5 shows that the inhibition of NADPH- and XO-mediated superoxide production by AVLE was comparable with the effect seen with SOD, a superoxide scavenger. It can thus be established that AVLE has a potent scavenging action against superoxide formation in both cell- and cell-free-mediated generation systems.

## 4. Discussion

A number of polyphenols including flavonol, flavonol glycosides, and flavan-3-ols have been identified in *Apocynum venetum* leaves extracts (AVLE) [20]. Regular intake of polyphenol-rich beverages and foods reduce the risk of cardiovascular diseases and its complications, mainly by reducing vascular oxidative stress and improving vasodilatation in blood vessels [8,21,22]. AVLE is a traditional Chinese medicinal herb widely available in China and Japan. This medicinal plant is gaining popularity as it is consumed as tea and herbal supplements to lower blood pressure. The present study demonstrates that AVLE induces a potent endothelium-dependent vasodilatation in isolated rat aortas and this response involved eNOS/NO/cGMP-mediated relaxation. Importantly, the present findings further indicate that AVLE-induced vasodilatation was associated with an increase of NO production via the Src/PI3K/Akt signalling pathway and it also exhibited potent antioxidant properties.

NO is released in response to vasodilators, such ACh and bradykinin, and is involved in several physiological processes including thrombosis formation, vasodilatation, vascular remodelling and smooth muscle cell proliferation [23]. Endogenous vasodilators stimulate the release of intracellular calcium (Ca^2+^) in endothelial cells and thereby activate NO synthesis by eNOS, which catalyzes the oxidation of L-arginine to NO and L-citrulline. After NO is synthesized, it diffuses rapidly to the vascular smooth muscle cells to reduce calcium influx inducing vasodilatation [24,25]. The present study showed that AVLE concentration-dependently induces relaxation in aortic rings pre-contracted with phenylephrine and the relaxation was abolished by L-NAME and ODQ, thus suggesting the role of eNOS-derived NO formation. In agreement with the previous finding, Kwan *et al.* [13] further demonstrated that AVLE-induced endothelium-dependent relaxation in rat aortas and released endothelium-derived hyperpolarizing factor in rat mesenteric arteries, which were then identified to be associated with activation of NO via the opening of the potassium channel and the release of NO plus EDHF, respectively.

eNOS activation and vasodilatation to blood flow induced shear stress and estrogens, and vascular endothelial growth factor may involve several kinase-dependent signalling pathways such as Src kinase, PI3K/Akt, protein kinase A (PKA), PKC or AMP-activated protein kinase [26,27,28]. The PI3K/Akt pathway is an important signalling pathway that has been indicated in the endothelial control of vascular tone by polyphenolic compounds. Red wine polyphenols have been shown to induce vasodilatation in blood vessels via activation of the PI3K/Akt pathway in endothelial cells by increasing intracellular Ca^2+^ levels which leads to phosphorylation of eNOS at Ser1177 and increasing NO formation [29]. In addition, red wine polyphenols effectively reduce the blood pressure in normo- and hypertensive rat models [30,31]. These cardiovascular protective effects of the polyphenols are attributable, at least in part, to an endothelium-dependent relaxation mechanism. Grapes are known to be the important ingredient of red wine, and a similar mechanism of action for the dilation of blood vessels have been observed with grape seed extracts, grape skin extracts and grape juice, which contain large amounts of polyphenols [9,10,32].

The present study demonstrated that inhibition of Src kinase and PI3K decreased endothelium-dependent relaxation of rat aortas to AVLE. Additionally, the NO-mediated vasorelaxation induced by AVLE was further confirmed through the measurement of total NO metabolites (NO_x_) in isolated rat aortas and HUVECs. AVLE at a concentration 10 µg/mL has been reported to exert potent endothelium-dependent relaxant effect due to the release of NO. We further demonstrated that AVLE at 10 µg/mL increased the total NO_x_ level in rat aortic tissues, and this increment was abolished in the presence of Src kinase and PI3K inhibitors, suggesting that the vasodilatation effect of AVLE in blood vessels are primarily through eNOS-derived NO production by the activation of Src/PI3K signalling pathways. Furthermore, in HUVECs, AVLE concentration dependently augmented the protein expression of phosphorylated Akt and eNOS. The phosphorylated expression of Akt and eNOS were increased in 5 min and remained elevated up to 30 min of exposure to AVLE (10 µg/mL). More importantly, we also found that the increase of expression of p-AKT^ser473^ and p-eNOS^ser1117^ and NO level by AVLE were remarkably reduced by Src kinase and PI3K inhibitors. The present findings are in line with the previous observations showing that AVLE effectively induced NO-mediated vasodilatation through the involvement of the Src/PI3K/Akt pathway.

In addition to stimulating NO production, the present finding has also demonstrated that AVLE possesses the ability to scavenge reactive oxygen species, superoxide anions, either in a cell- or cell-free environments to improve NO bioavailability. Superoxide anions rapidly oxidize the highly-reactive NO into form the toxic peroxynitrite which, in turn, uncouples eNOS by oxidizing the essential NOS-redox-sensitive co-factor tetrahydrobiopterin and causes eNOS to produce more superoxide anion. This continuous cascade of events reduces the bioavailability of NO [33,34]. Kwan *et al.* [12] have reported that AVLE, unlike carbachol, has a long-lasting vasorelaxant effect and postulated that the active ingredients of AVLE may contain superoxide anion scavengers or inhibitors of the superoxide anion-generating system, thus permitting more effective and longer generation of NO, which would otherwise be degraded by coproduced superoxide anions. Previously, we demonstrated that AVLE inhibits Ang II-induced contraction via its superoxide anion scavenging and NO releasing effect [35]. Polyphenols have been known to cause a long lasting activation of eNOS through its phosphorylation activity whilst its antioxidant activity may reduce NO degradation and further prolong its half-life [8,29].

## 5. Conclusions

AVLE is a potent vasodilator through its endothelium-dependent NO releasing action which is mediated by an upstream Src/PI3K/Akt signalling pathway. The vasoprotective effect of AVLE is also associated with increased NO bioavailability in endothelial cells and it is likely attributable, at least in part, to its superoxide scavenging activity. Hence, the current study has provided further cellular insight to the vasoprotective effect of AVLE and reinforces the use of this traditional medicinal herb in the treatment of hypertension.
